# Integrative analysis of plasma and substantia nigra in Parkinson’s disease: unraveling biomarkers and insights from the lncRNA–miRNA–mRNA ceRNA network

**DOI:** 10.3389/fnagi.2024.1388655

**Published:** 2024-05-09

**Authors:** Ka-Yoon Chun, Seung-Nam Kim

**Affiliations:** College of Korean Medicine, Dongguk University, Goyang, Republic of Korea

**Keywords:** Parkinson’s disease, microRNA, lncRNA, ceRNA network, biomarker, GEO database, meta-analysis, bioinformatics analysis

## Abstract

**Introduction:**

Parkinson’s disease (PD) is a rapidly growing neurological disorder characterized by diverse movement symptoms. However, the underlying causes have not been clearly identified, and accurate diagnosis is challenging. This study aimed to identify potential biomarkers suitable for PD diagnosis and present an integrative perspective on the disease.

**Methods:**

We screened the GSE7621, GSE8397-GPL96, GSE8397-GPL97, GSE20163, and GSE20164 datasets in the NCBI GEO database to identify differentially expressed (DE) mRNAs in the substantia nigra (SN). We also screened the GSE160299 dataset from the NCBI GEO database to identify DE lncRNAs and miRNAs in plasma. We then constructed 2 lncRNA–miRNA–mRNA competing endogenous RNA (ceRNA) regulatory networks based on the ceRNA hypothesis. To understand the biological function, we performed Kyoto Encyclopedia of Genes and Genomes pathway and Gene Ontology analyses for each ceRNA network. The receiver operating characteristic analyses (ROC) was used to assess ceRNA results.

**Results:**

We identified 7 upregulated and 29 downregulated mRNAs as common DE mRNAs in the 5 SN datasets. In the blood dataset, we identified 31 DE miRNAs (9 upregulated and 22 downregulated) and 332 DE lncRNAs (69 upregulated and 263 downregulated). Based on the determined interactions, 5 genes (P2RX7, HSPA1, SLCO4A1, RAD52, and SIRT4) appeared to be upregulated as a result of 10 lncRNAs sponging 4 miRNAs (miR-411, miR-1193, miR-301b, and miR-514a-2/3). Competing with 9 genes (ANK1, CBLN1, RGS4, SLC6A3, SYNGR3, VSNL1, DDC, KCNJ6, and SV2C) for miR-671, a total of 26 lncRNAs seemed to function as ceRNAs, influencing genes to be downregulated.

**Discussion:**

In this study, we successfully constructed 2 novel ceRNA regulatory networks in patients with PD, including 36 lncRNAs, 5 miRNAs, and 14 mRNAs. Our results suggest that these plasma lncRNAs are involved in the pathogenesis of PD by sponging miRNAs and regulating gene expression in the SN of the brain. We propose that the upregulated and downregulated lncRNA-mediated ceRNA networks represent mechanisms of neuroinflammation and dopamine neurotransmission, respectively. Our ceRNA network, which was associated with PD, suggests the potential use of DE miRNAs and lncRNAs as body fluid diagnostic biomarkers. These findings provide an integrated view of the mechanisms underlying gene regulation and interactions in PD.

## 1 Introduction

Parkinson’s disease (PD) is one of the most rapidly growing neurological disorders and is characterized by movement symptoms, such as rigidity, slowness, and tremor (Emamzadeh and Surguchov, 2018; Armstrong and Okun, 2020; Morelli and Pinna, 2023). These motor symptoms are caused by the pathological conditions of dopaminergic neurons in the substantia nigra (SN), which includes α-synuclein (SNCA) aggregation, oxidative stress, ferroptosis, mitochondrial dysfunction, and neuroinflammation (Surmeier, 2018; Dong-Chen et al., 2023). However, since these symptoms vary widely, and their underlying causes have not been clearly identified, they may be not specific to PD, emphasizing the need for an accurate diagnosis (Madabushi et al., 2023). Recent studies have indicated that genetic factors such as SNCA, parkin (PRKN), PTEN-induced kinase 1 (PINK1), leucine-rich repeat kinase 2 (LRRK2), and vacuolar protein sorter-35 (VPS35) play crucial roles in PD (Funayama et al., 2023). Despite substantial progress in genetic development, body fluid diagnostic biomarkers for PD are still lacking (Adam et al., 2023). Consequently, the identification of potential biomarkers suitable for diagnosis continues to pose challenges.

Given that protein-coding genes constitute only 2% of the human genome, non-coding RNAs (ncRNAs), which are transcripts that do not code for any protein, are considered key regulatory molecules in all cellular processes (Ranga et al., 2023). Among the ncRNAs, microRNAs (miRNAs) and long non-coding RNAs (lncRNAs) are potential biomarkers of PD (Kuo et al., 2021). However, a single biomarker cannot fully elucidate the pathological mechanism of PD and fails to attain the high specificity and sensitivity required for accurate diagnosis (Cocco et al., 2023). Numerous studies on PD have proposed integrated biomarkers based on the competing endogenous RNA (ceRNA) hypothesis, which explains the competition between coding RNAs (mRNAs) and ncRNAs for shared miRNAs (Lin et al., 2021). Dysregulation of ceRNA networks has been observed in PD, influencing apoptosis, SNCA misfolding, mitochondrial dysfunction, autophagy, and neuroinflammation, all implicated in PD pathogenesis (Asadi et al., 2023). For example, LINC-p21 alters apoptosis in SH-SY5Y cells by sponging miR-1277-5p (Xu et al., 2018). LINC00943 alleviates neuronal damage in PD models by sponging miR-7-5p and increasing CXCL12 expression (Lian et al., 2021). Furthermore, two ceRNA axes, LINC09238/miR-30c-5p/LRRK2 and LINC001128/miR-30c-5p/ATP13A2, are involved in PD through the regulation of mitochondrial dysfunction (Yousefi et al., 2022). However, these studies have reported different single ceRNA axes, and diagnostic biomarkers for PD remain questionable.

In this study, we constructed an lncRNA–miRNA–mRNA ceRNA regulatory network to reveal the impact of lncRNA–miRNA competition in the blood on SN mRNA. To propose an integrative perspective on PD, we collected several datasets from the Gene Expression Omnibus (GEO) database and analyzed them using various bioinformatics software tools.

## 2 Materials and methods

**Figure 1:**
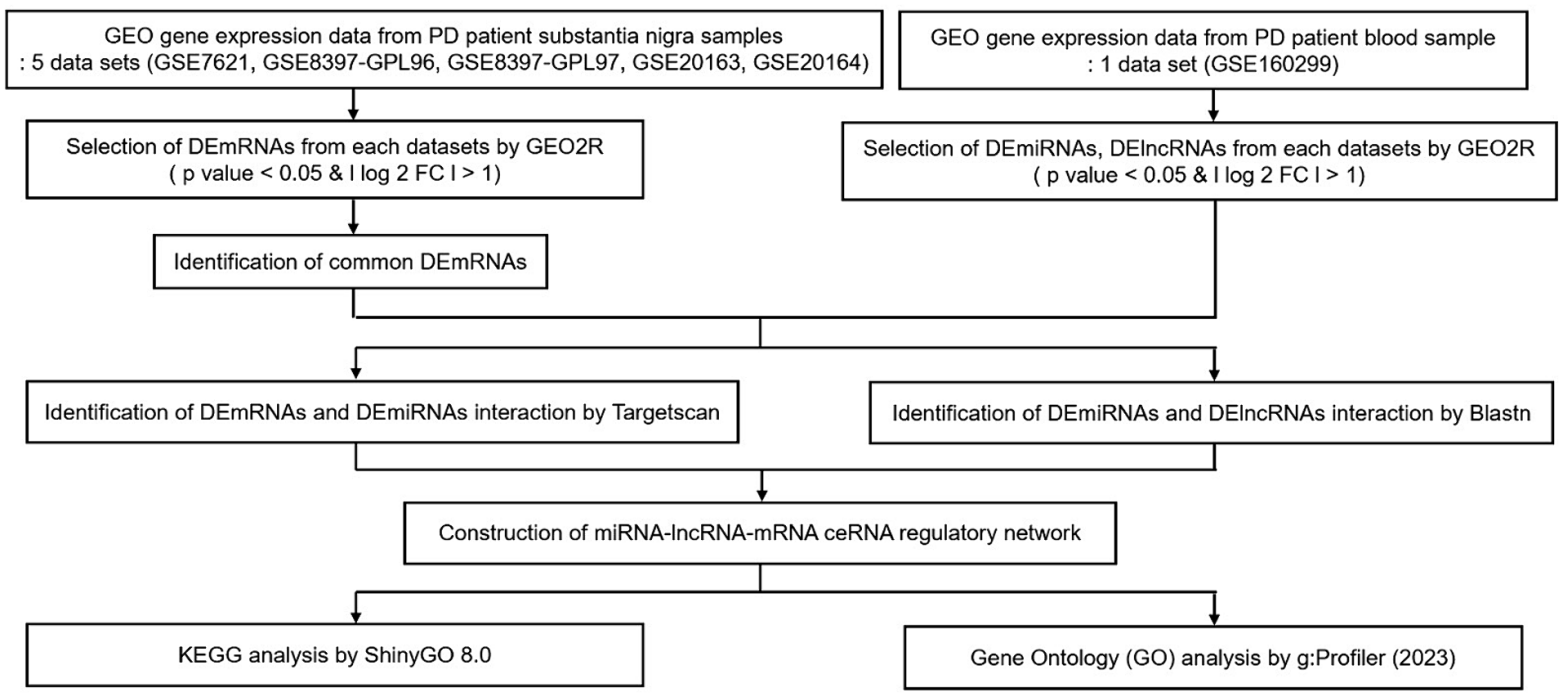
Flow chart of our study. Data collection, processing, construction of ceRNA network, and KEGG & GO analysis. GEO, gene expression omnibus; PD, Parkinson’s disease; DE, differential expression; FC, fold change; KEGG, Kyoto Encyclopedia of Genes and Genomes.

Our aim was to construct lncRNA–miRNA–mRNA ceRNA networks according to the flowchart shown in Figure 1.

### 2.1 Data collection of patients with PD

In this study, “Parkinson’s disease” was used as a keyword to search for microarray data in the GEO^1^ of the National Center for Biotechnology Information (NCBI). The following inclusion criteria were used to select the dataset for analysis: (1) studies on the SN in patients with PD in which the experiment type was expression profiling by array, and (2) studies on blood in patients with PD in which the experiment type was expression profiling by high-throughput sequencing or ncRNA profiling, due to the determination of miRNA and lncRNA expression data. Datasets with no significant differentially expressed (DE) mRNAs, miRNAs, or lncRNAs were excluded.

We selected five datasets from SN studies (GSE7621, GSE8397-GPL96, GSE8397-GPL97, GSE20163, and GSE20164) and one dataset from blood studies (GSE160299). Information on the microarray datasets is presented in Table 1. Only two blood studies were registered in the database, of which only one was selected because no significantly expressed miRNAs or lncRNAs were found in the other. To assess their associations with PD, principal component analysis (PCA) was conducted between PD patients and healthy controls for each dataset using the R statistical language version 4.3.1.

**Table 1:** Datasets selected in our study.

Tissue	GEO accession	Platform	Sample	Types	Up / Down
SN (Substantia Nigra)	GSE7621	GPL570	PD(16) / CON(9)	mRNA	688 (↑) / 450 (↓)
	GSE8397-GPL96	GPL96	PD(24) / CON(15)	mRNA	62 (↑) / 256 (↓)
	GSE8397-GPL97	GPL97	PD(24) / CON(15)	mRNA	19 (↑) / 182 (↓)
	GSE20163	GPL96	PD(8) / CON(9)	mRNA	231 (↑) / 165 (↓)
	GSE20164	GPL96	PD(6) / CON(5)	mRNA	364 (↑) / 210 (↓)
Plasma	GSE160299	GPL20301	PD(3) / CON(4)	miRNA lncRNA	9 (↑) / 22 (↓) 69 (↑) / 263 (↓)

### 2.2 Data preprocessing and identification of DE miRNAs, lncRNAs, and mRNAs

We used GEO2R online software to process each dataset to compare healthy controls and PD patient samples. The *p*-values and false discovery rate (FDR) were calculated using the *t*-test and Benjamini and Hochberg test, respectively. The statistical significance of selecting DE genes between healthy controls and PD patient samples was set to *p* < 0.05 and | Log 2 Fold Change | > 1. Probes with no annotated genes were excluded from the analysis. The probe with the highest fold-change value was selected if multiple probes matched the same gene symbol.

### 2.3 Meta-analysis to identify common DE mRNAs in datasets from brain SN

Using vote-counting generic ways of combining information (Ramasamy et al., 2008), a meta-analysis was performed on five datasets from the brain SN. For comparison, the results were visualized using Venn diagrams and heatmaps using R statistical language 4.3.1. Subsequently, DE mRNAs that were common to at least three datasets were defined as common DE mRNAs and subjected to further analysis.

### 2.4 Identification of DE mRNA–DE miRNA and DE lncRNA–DE miRNA interactions

To examine the interactions between common DE mRNAs and DE miRNAs, we collected the predicted miRNA targets of common DE mRNAs using TargetScan 8.0.^2^ We then identified common DE mRNAs that contained DE miRNAs for the predicted targets, and these mRNAs were used to construct the network. Genes without predicted targets in the database were excluded.

To identify the interactions between DE lncRNAs and DE miRNAs, we retained only miRNAs that were present in both DE miRNAs from the blood dataset and the targeting miRNAs of common DE mRNAs from the SN datasets. To predict miRNA-binding sites in DE lncRNAs, we used Blastn online software, which identifies regions of similarity between biological sequences.

### 2.5 Construction of the lncRNA–miRNA–mRNA ceRNA regulatory network

Because lncRNAs regulate the expression of mRNA by sponging miRNAs, upregulated or downregulated miRNAs and lncRNAs, or mRNAs that were inversely related to miRNAs in the miRNA–mRNA and lncRNA–miRNA interaction pairs, were chosen to construct the lncRNA–miRNA–mRNA ceRNA network (Xu et al., 2019). To construct lncRNA-mediated ceRNA networks in PD, we used Cytoscape software 3.9.1 (Shannon et al., 2003).

### 2.6 Kyoto encyclopedia of genes and genomes (KEGG) pathway and gene ontology (GO) analysis of target genes in the ceRNA network

To understand the biological functions, we performed KEGG pathway and GO analyses of the coding genes involved in the constructed ceRNA network. For KEGG pathway analysis and visualization, we used ShinyGO 0.80 (Luo and Brouwer, 2013; Ge et al., 2020; Kanehisa et al., 2021). To search for significantly enriched GO terms in biological processes (BP), cellular components (CC), and molecular functions (MF), G:Profiler (2023) was used to identify the top 10 GO terms (Kolberg et al., 2023). GO terms were interpreted with reference to AmiGO and classified as the best descriptive terms (Carbon et al., 2009). Both the KEGG pathway and GO analyses were considered significantly enriched, with an FDR cutoff of 0.05.

### 2.7 ROC analysis

To assess the accuracy of ceRNA results as a diagnostic marker, we conducted receiver operating characteristic (ROC) curve analysis and calculated the area under the curve (AUC) using a webtool from easyROC.

## 3 Results

### 3.1 Meta-analysis of common DE mRNAs in datasets from brain SN and identification of DE miRNAs and DE lncRNAs

**Figure 2:**
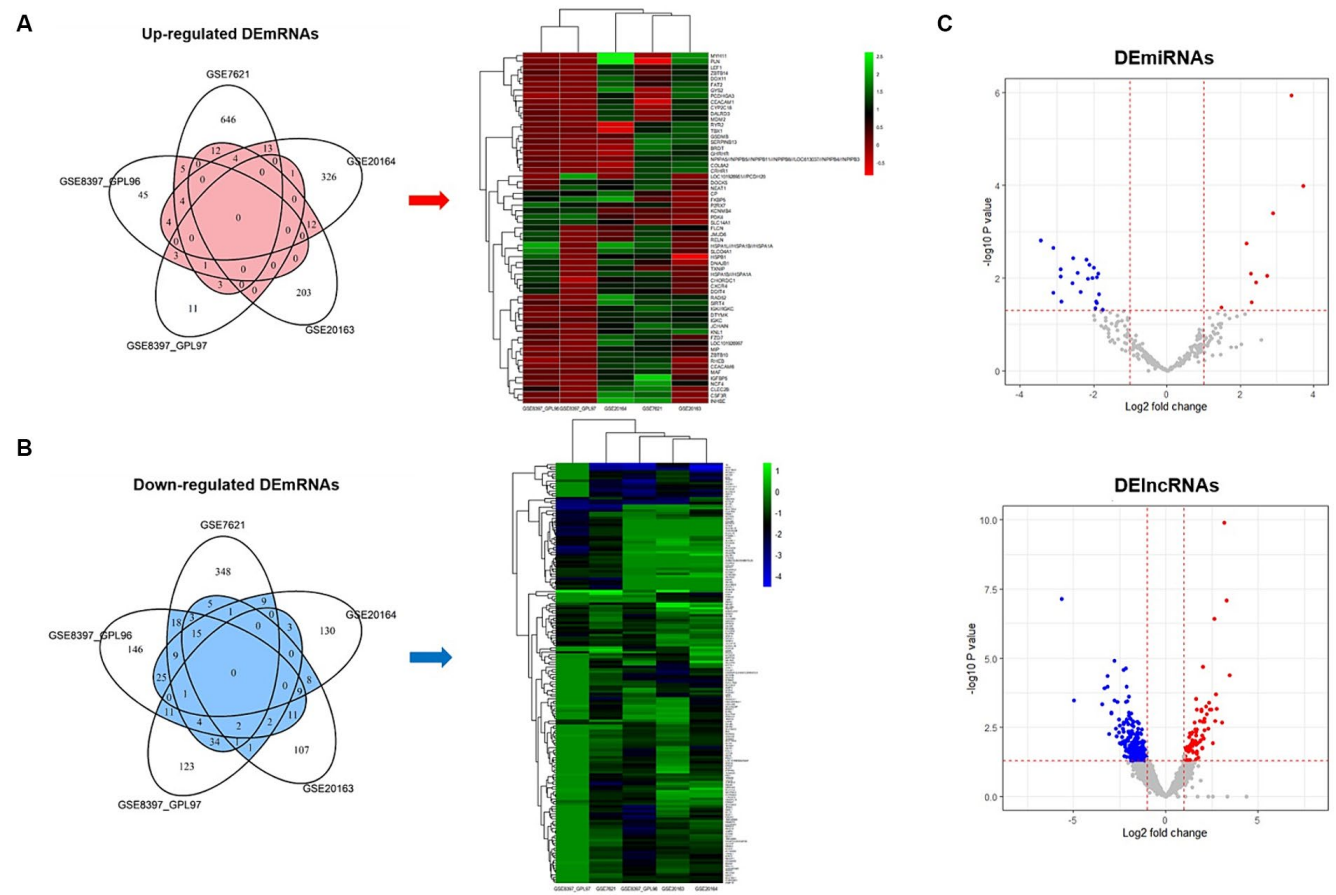
Venn diagrams, heatmap plots, and volcano plots of differentially expressed mRNAs, miRNAs, and lncRNAs. **(A)** Venn diagram and heatmap plot of upregulated mRNAs. Genes belong to the red area of the Venn diagram used for heatmap analysis. **(B)** Venn diagram and heatmap plot of downregulated mRNAs. Genes belong to the blue area of the Venn diagram used for heatmap analysis. **(C)** Volcano plot of differentially expressed miRNAs and lncRNAs. Red indicates upregulated and blue indicates downregulated.

We used five datasets from brain SN studies to perform meta-analyses to screen for DE mRNAs that were common between patients with PD and healthy controls. The association of each dataset with PD was confirmed through PCA plots (Supplementary Figure 1). To identify common DE mRNAs across these datasets, the DE mRNAs in each dataset were demonstrated using Venn diagrams and heatmap plots (Figures 2A,B), which showed 7 upregulated and 29 downregulated DE mRNAs in more than three datasets. Volcano plots showing the expression of miRNAs and lncRNAs with *p* < 0.05 and | Log 2 Fold Change | > 1 were screened for further analysis (Figure 2C), which revealed 31 DE miRNAs (9 upregulated and 22 downregulated) and 332 DE lncRNAs (69 upregulated and 263 downregulated).

### 3.2 Identification of DE miRNA–DE mRNA and DE miRNA–DE lncRNA interactions

Based on the relationships in ceRNA theory, we searched for miRNA–mRNA and miRNA–lncRNA interactions using Targetscan and Blastn, respectively. We identified 7 miRNA–mRNA and 19 miRNA–lncRNA interactions in the upregulated ceRNA network (Table 2A). For the downregulated ceRNA network, we identified 9 miRNA–mRNA and 26 miRNA–lncRNA interactions (Table 2B).

**Table 2A:** Interactions between downregulated miRNAs and upregulated mRNAs or lncRNAs.

miRNAs from plasma	mRNAs from SN	miRNA-mRNA interaction score	lncRNAs from plasma	miRNA-lncRNA interaction score
miR-411	P2RX7 HSPA1	−0.13 −0.02	LINC00887 HILPDA-AS1 SLC9A3R1-AS1 DARS1-AS1	3.1 0.6 0.81 0.029
miR-1193	P2RX7 SLCO4A1	−0.17 −0.3	LINC00887 HILPDA-AS1 SLC9A3R1-AS1	0.63 0.031 0.65
miR-301b	RAD52	−0.19	LINC00887 HILPDA-AS1 SLC9A3R1-AS1 MSC-AS1 YEATS2-AS1 LINC00861 LINC02605 LINC02576	0.63 0.12 0.026 0.12 0.047 0.019 0.086 0.11
miR-514a-2/3	RAD52 SIRT4	−0.18 −0.19	LINC00887 HILPDA-AS1 COL4A2-AS2 LINC02576	0.72 0.55 0.051 0.0008

The interaction score between miRNA and mRNA was evaluated using the Total context++ score from Targetscan, and the interaction score between miRNA and lncRNA was evaluated using E value from Blastn.

**Table 2B:** Interactions between upregulated miRNAs and downregulated mRNAs or lncRNAs.

miRNAs from plasma	mRNAs from SN	miRNA-mRNA interaction score	lncRNAs from plasma	miRNA-lncRNA interaction score
miR-671	ANK1 CBLN1 RGS4 SLC6A3 SYNGR3 VSNL1 DDC KCNJ6 SV2C	−0.10 −0.06 −0.08 −0.17 −0.30 −0.60 −0.27 −0.26 −0.02	LINC02179 LINC01762 GALNT9-AS1 HOXB-AS3 LINC01238 LINC02519 LINC02675 LINC02816 LINC01345 LINC01756 LINC01657 LINC02572 SEMA3B-AS1 PLCXD2-AS1 LINC01487 LINC02428 LINC01951 LINCMD1 LINC02527 LINC03015 LINC02735 GPC5-AS1 SPATA8-AS1 LINC02876 LINC01987 LINC00322	0.048 0.022 0.015 0.002 0.008 0.048 0.041 0.006 0.013 0.008 0.0002 0.019 0.005 0.013 0.019 0.019 0.028 0.005 0.002 0.027 0.003 0.018 0.023 0.039 0.009 0.003

The interaction score between miRNA and mRNA, as well as between miRNA and lncRNA, were evaluated using the same method as described in Table 2A.

### 3.3 Construction of the lncRNA–miRNA–mRNA ceRNA network

**Figure 3:**
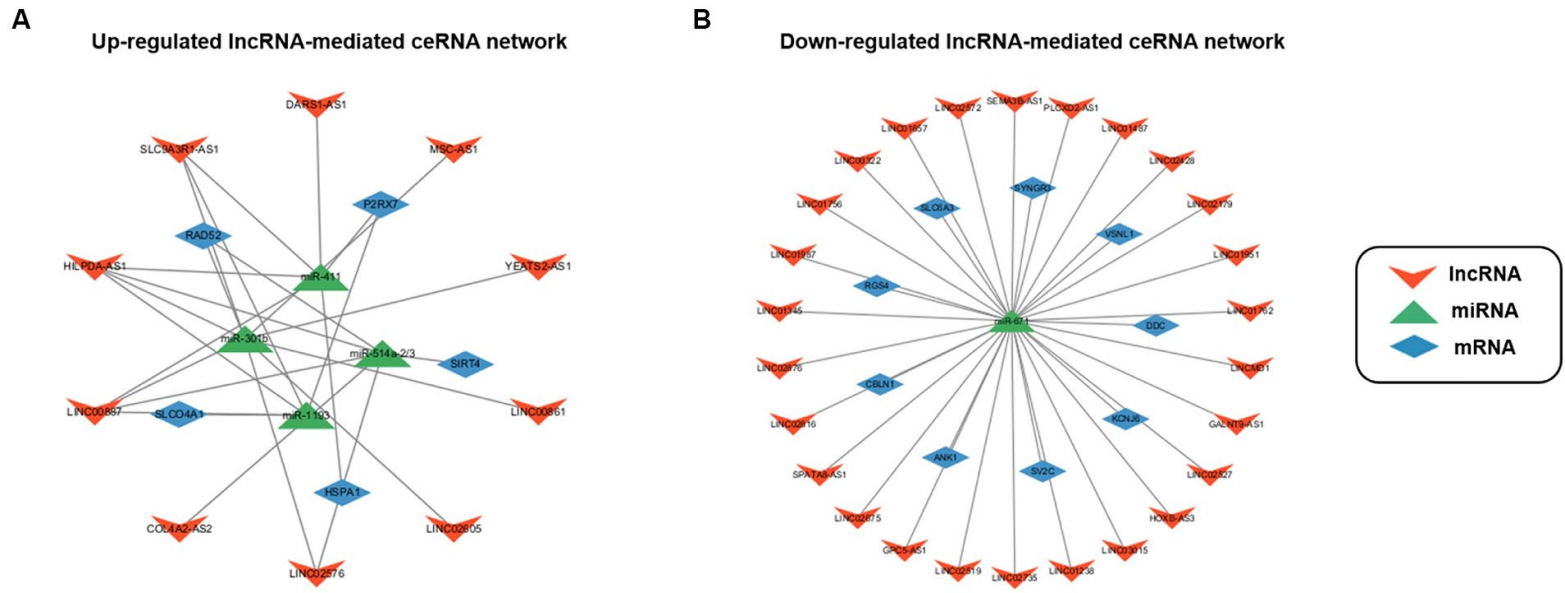
Construction of lncRNA–miRNA–mRNA ceRNA regulatory network. **(A)** The lncRNA-mediated upregulated ceRNA network. **(B)** The lncRNA-mediated downregulated ceRNA network. Red, green, and blue indicate lncRNA, miRNA, and mRNA, respectively.

Based on these interactions, we constructed an lncRNA–miRNA–mRNA ceRNA regulatory network. As a result of the 10 lncRNAs sponging 4 miRNAs (miR-411, miR-1193, miR-301b, and miR-514a-2/3), 5 genes (P2RX7, HSPA1, SLCO4A1, RAD52, and SIRT4) appeared to be upregulated (Figure 3A). Competing with 9 genes (ANK1, CBLN1, RGS4, SLC6A3, SYNGR3, VSNL1, DDC, KCNJ6, and SV2C) for miR-671, a total of 26 lncRNAs seemed to function as ceRNAs, influencing genes to be downregulated (Figure 3B).

### 3.4 KEGG pathway and GO enrichment analyses of DE mRNAs in the ceRNA network

**Figure 4:**
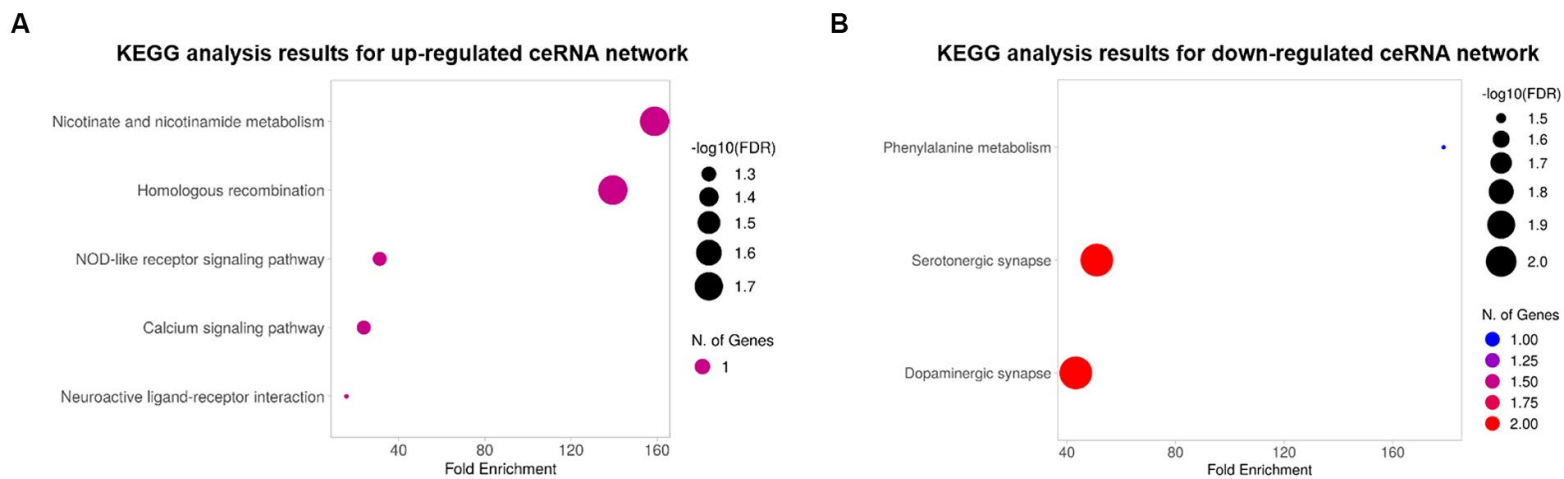
The bubble plots of KEGG pathway analysis results. **(A)** Results for the upregulated ceRNA network. **(B)** Results for the downregulated ceRNA network. The color and size of bubbles indicate the number of genes associated with each pathway and the -log 10 (FDR), respectively.

To explore the biological functions of the ceRNA network, KEGG pathway and GO enrichment analyses were performed with an FDR cutoff of 0.05. According to KEGG pathway analysis, DE mRNAs in the upregulated ceRNA network were involved in “nicotinate and nicotinamide metabolism,” “homologous recombination,” “NOD-like receptor signaling pathway,” “calcium signaling pathway,” and “neuroactive ligand-receptor interaction” (Figure 4A). DE mRNAs in downregulated ceRNA networks were relevant to “Phenylalanine metabolism,” “Serotonergic synapse,” and “Dopaminergic synapse” (Figure 4B).

GO analyses were also conducted with DE mRNAs in each lncRNA-mediated ceRNA network associated with BP, CC, and MF. The top 10 enriched GO terms in each of the three major categories were selected based on the FDR (Tables 3A, 3B). These results indicate that genes associated with the upregulated ceRNA network are related to nicotinamide adenine dinucleotide (NAD+) and the transport of eicosanoids such as prostaglandins. Genes linked to the downregulated ceRNA network were associated with secretion, the biosynthetic process of catecholamines (such as dopamine), and synapses that use dopamine as a neurotransmitter and transmit signals to the postsynaptic membrane.

**Table 3A:** The top 10 GO enriched terms of the upregulated lncRNA-mediated ceRNA network.

Source	Term_name	Term_id	Adjusted *p*_value	Term_size	Query_size	Intersections
GO:MF	lipoamidase activity	GO:0061690	0.049904	1	4	SIRT4
GO:MF	NAD-dependent protein lipoamidase activity	GO:0106419	0.049904	1	4	SIRT4
GO:MF	NAD-dependent protein biotinidase activity	GO:0106420	0.049904	1	4	SIRT4
GO:BP	Prostaglandin transport	GO:0015732	0.007445	29	4	P2RX7, SLCO4A1
GO:BP	Eicosanoid transport	GO:0071715	0.038055	65	4	P2RX7, SLCO4A1

**Table 3B:** The top 10 GO enriched terms of the downregulated lncRNA-mediated ceRNA network.

Source	Term_name	Term_id	Adjusted *p*_value	Term_size	Query_size	Intersections
GO:BP	Secretion	GO:0046903	0.000749	954	9	SYNGR3, VSNL1, SV2C, ANK1, CBLN1, SLC6A3
GO:BP	Exocytosis	GO:0006887	0.009922	346	9	SYNGR3, VSNL1, SV2C, ANK1
GO:BP	Secretion by cell	GO:0032940	0.011568	821	9	SYNGR3, VSNL1, SV2C, ANK1, CBLN1
GO:BP	Dopamine biosynthetic process	GO:0042416	0.012491	12	9	DDC, SLC6A3
GO:BP	Export from cell	GO:0140352	0.016861	887	9	SYNGR3, VSNL1, SV2C, ANK1, CBLN1
GO:BP	Catecholamine biosynthetic process	GO:0042423	0.039663	21	9	DDC, SLC6A3
GO:BP	Catechol-containing compound biosynthetic Process	GO:0009713	0.039663	21	9	DDC, SLC6A3
GO:CC	Dopaminergic synapse	GO:0098691	0.001641	14	9	SV2C, SLC6A3
GO:CC	Synapse	GO:0045202	0.014997	1,451	9	SYNGR3, SV2C, ANK1, CBLN1, SLC6A3

### 3.5 ROC analysis of DE miRNAs and DE lncRNAs in the ceRNA network

**Figure 5:**
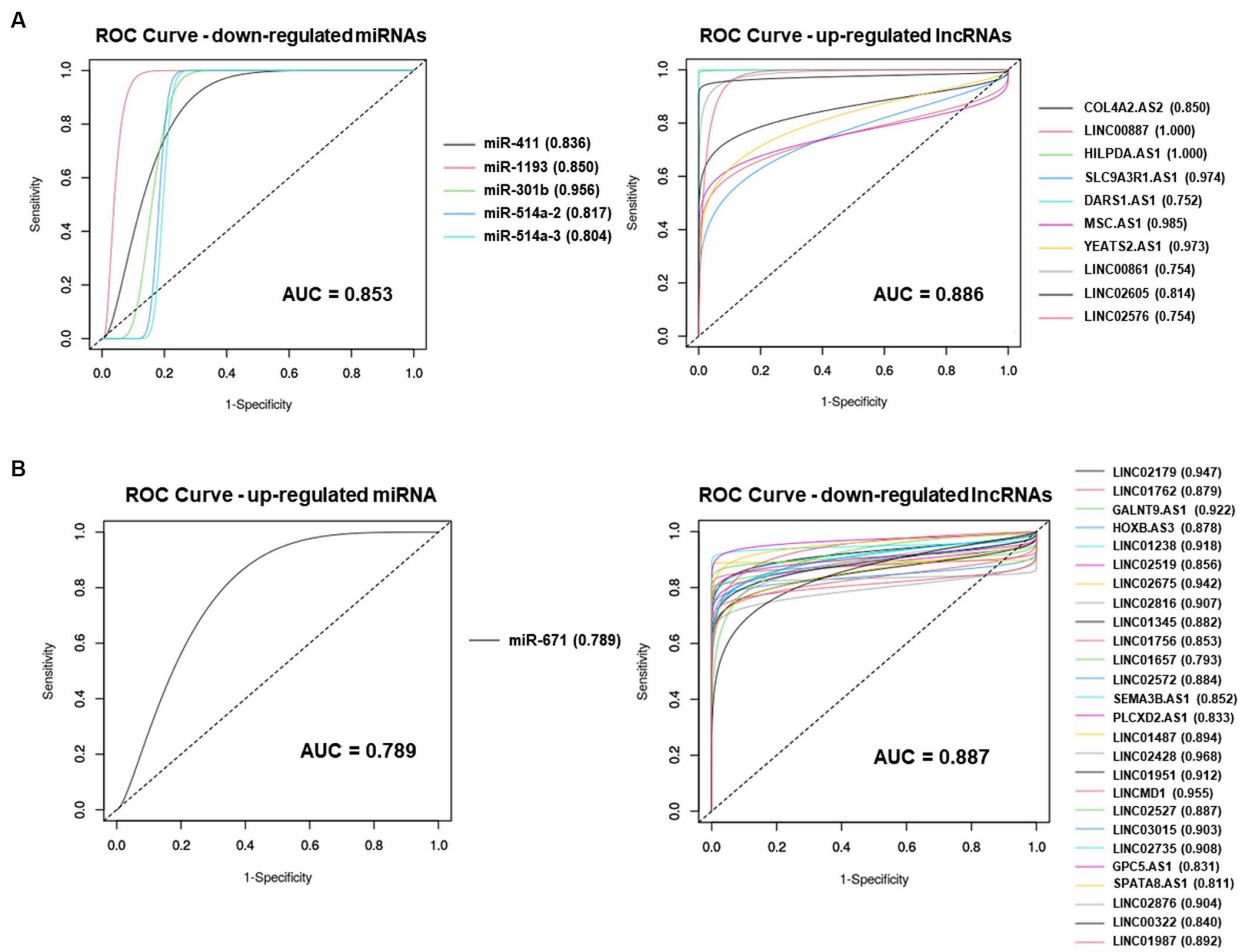
The receiver operating characteristic (ROC) curve analysis results for each ceRNA network. **(A)** ROC curve of DE miRNAs and DE lncRNAs on upregulated ceRNA network. **(B)** ROC curve of DE miRNAs and DE lncRNAs on downregulated ceRNA network.

Furthermore, we performed ROC analysis for each ceRNA network to evaluate the accuracy of ceRNA results as a diagnostic marker. Downregulated miRNAs and upregulated lncRNAs in upregulated ceRNA network showed high accuracy, with AUC values of 0.853 and 0.886, respectively (Figure 5A). Similarly, upregulated miRNA and downregulated lncRNAs in downregulated ceRNA network also demonstrated high accuracy, with AUC vaules of 0.789 and 0.887, respectively (Figure 5B).

## 4 Discussion

Recently, an increasing number of studies have identified PD-specific genes in the brain, including SNCA, PRKN, PINK1, and LRRK2. However, it is problematic to use these genes as body fluid diagnostic biomarkers, and the quest for diagnostic biomarkers using body fluids remains challenging. As ncRNAs, including lncRNAs and miRNAs, have emerged as key factors in PD, we focused on the competing regulatory networks between ncRNAs to propose an integrative perspective on PD.

In this study, we constructed two lncRNA-mediated ceRNA networks based on the expression profiles of patients with PD. We screened out common DE mRNAs from the GEO database to investigate genes expressed in the brain SN, as well as DE lncRNAs and DE miRNAs to include genes expressed in plasma. To check their associations with PD, we also demonstrated the difference between PD patients and healthy controls using PCA plots. Subsequently, we constructed two lncRNA–miRNA–mRNA ceRNA regulatory networks to elucidate the effect of plasma lncRNAs on changes in SN mRNA. We conducted KEGG pathway and GO enrichment analyses to comprehend the biological functions of each ceRNA network. Finally, we performed ROC analysis to evaluate the accuracy of ceRNA results as a diagnostic marker.

In the upregulated lncRNA-mediated ceRNA network, 10 lncRNAs were found to regulate the expression of 5 genes in the SN by sponging 4 miRNAs. Several nodes in this ceRNA network have been shown to play crucial roles in PD associated with neuroinflammation. For example, P2RX7 has been widely reported to play a central role in PD models by promoting neuroinflammation (Calzaferri et al., 2020). HSPA1, also known as Hsp70, has been found to be released from damaged cells, acting as a local danger signal (Giuliano et al., 2011). Both SIRT4 and RAD52 have received attention for their therapeutic effects on DNA damage, which has been found to induce inflammatory responses in several neuronal cell types (Wang et al., 2021, 2023). Although these genes have contradictory effects, neuroinflammation induces damage to nerve tissues while promoting the activation of neuroprotective mechanisms (Gelders et al., 2018). In addition, our KEGG pathway and GO enrichment analyses showed that the genes in the upregulated lncRNA-mediated ceRNA network were significantly enriched in nicotinamide metabolism and prostaglandin transport. These pathways have been reported to regulate neuroinflammation during the development of PD (Corwin et al., 2018; Zhou et al., 2018). Therefore, it is reasonable to hypothesize that this upregulated lncRNA-mediated ceRNA network may explain the neuroinflammatory mechanisms in patients with PD.

In a downregulated lncRNA-mediated ceRNA network, 26 lncRNAs appeared to be downregulated, leading to the downregulation of 9 genes in the SN by miR-671. Several nodes in this ceRNA network have been studied for their crucial roles in PD. For example, the brain expression of SLC6A3 (Habak et al., 2014), SYNGR3 (Ho et al., 2023), DDC (Pereira et al., 2023), and SV2C (Dunn et al., 2017) has been shown to modulate dopamine neurotransmission in PD models. Additionally, based on our KEGG pathway and GO enrichment analysis results, genes in the downregulated lncRNA-mediated ceRNA network were significantly enriched in the dopaminergic synapses. Therefore, we speculate that this downregulated lncRNA-mediated ceRNA network plays an important role in patients with PD by regulating dopamine neurotransmission.

Our study had some limitations. First, only one dataset from PD patient blood was available in the GEO database. Second, the functions of the ncRNAs in each ceRNA network have not been previously reported in association with PD, or they showed different expression trends compared to our study. Some studies have indicated that miR-671 is downregulated to play a role in MPP + −induced SK-N-SH cells (Zhang et al., 2022; Hao et al., 2023). However, these studies were conducted at the cellular level, and it remains unclear how miR-671 in the blood affects the SN in the brain. Similarly, the effects of other RNA species on brain regions might also require further investigation. Thus, it is more reasonable to propose that the ceRNA regulatory network might be implicated in the pathogenesis of PD through the function of mRNAs, and that miRNAs and lncRNAs are significant body fluid biomarkers. Further experiments are needed to reveal the exact functions of blood ncRNAs in PD.

Despite these limitations, our study holds significance as it persevered in the analysis of blood datasets, which is in contrast to previous studies that relied solely on bioinformatics tools offering reported ncRNA data. By incorporating analyses of blood samples from patients with PD, future research has the potential to provide a more meaningful ceRNA network. Such efforts are likely to contribute to a more precise elucidation of the pathological mechanisms of PD, ultimately enhancing treatment strategies.

In conclusion, we constructed a ceRNA regulatory network in patients with PD, including 36 lncRNAs, 5 miRNAs, and 14 mRNAs. Our results suggest that these lncRNAs (from the plasma) are involved in PD pathogenesis by sponging miRNAs and regulating gene expression in the SN of the brain. We propose that the upregulated lncRNA-mediated ceRNA network may explain the mechanism underlying neuroinflammation in PD. In addition, the downregulated lncRNA-mediated ceRNA network appeared to contribute to PD by influencing dopaminergic neurotransmission. As our ceRNA network has been found to be associated with PD, the DE miRNAs and lncRNAs in our ceRNA network could be utilized as body fluid diagnostic biomarkers. Our findings provide an integrated view of the mechanisms underlying gene regulation and interactions in PD.

## Data availability statement

Publicly available datasets were analyzed in this study. This data can be found here: five datasets from SN studies (GSE7621, GSE8397-GPL96, GSE8397-GPL97, GSE20163, and GSE20164) and one dataset from blood studies (GSE160299).

## Author contributions

K-YC: Conceptualization, Formal analysis, Investigation, Visualization, Writing – original draft. S-NK: Conceptualization, Supervision, Writing – review & editing.

## Footnotes

^1^ http://www.ncbi.nlm.nih.gov/gds/

^2^ https://www.targetscan.org/vert_80/

## Supplementary material

FIGURE S1 The principal component analysis (PCA) for each GEO datasets. Each of **(A–E)** indicates GSE7621, GSE8397-GPL96, GSE8397-GPL97, GSE20163, and GSE20164, respectively.
