# Supplementary figures and images for: Integrative analysis of plasma and substantia nigra in Parkinson’s disease: unraveling biomarkers and insights from the lncRNA–miRNA–mRNA ceRNA network
Source: Front Aging Neurosci. 2024 May 9;16:1388655. doi: 10.3389/fnagi.2024.1388655 (PMC11112011; doi:10.3389/fnagi.2024.1388655)

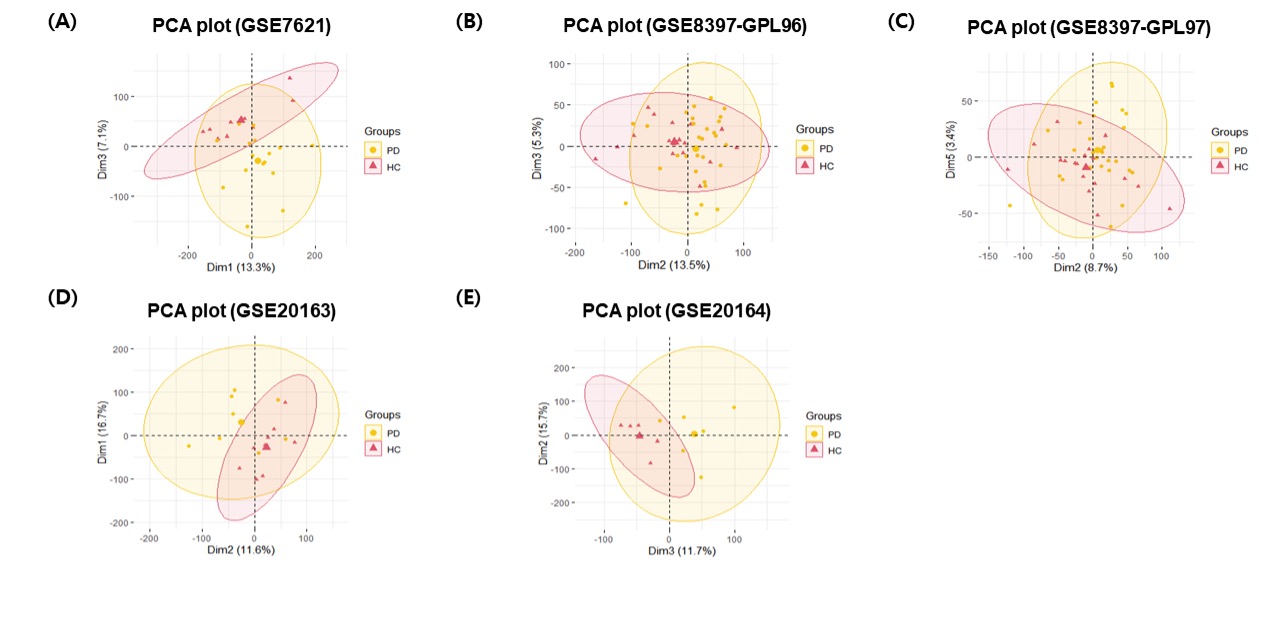

Supplement: FIGURE S1 — The principal component analysis (PCA) for each GEO datasets. Each of (A–E) indicates GSE7621, GSE8397-GPL96, GSE8397-GPL97, GSE20163, and GSE20164, respectively. [file Image_1.JPEG]
